# Supplementary material for: Association between tumor mutation profile and clinical outcomes among Hispanic Latina women with triple-negative breast cancer
Source: PLoS One. 2020 Sep 4;15(9):e0238262. doi: 10.1371/journal.pone.0238262 (PMC7473586; doi:10.1371/journal.pone.0238262)
Supplement: Attachment — Submitted filename: Philipovskiy-Response_reviewer_2_final.docx [file pone.0238262.s001.docx]

**Response to reviewer # 2**

Thank you very much for your positive feedback on our paper. We greatly appreciate the time you have spent in your careful review of our manuscript. We are grateful for your thoughtful comments and constructive suggestions, which have greatly improved the quality of this paper. Please find our responses below (***reviewers’ comments are in*** ***italics***):

*Reviewer #2: This is a very important and timely manuscript. The tumor mutation profiles and outcomes among Hispanic women with triple-negative breast cancer (TNBC) is under studied. Authors put together a very good manuscript using Hispanic-Latina (HL) TNBC patients data. I do have some comments and suggestions to make this manuscript comprehensive. These are as follows:*

*Abstract:
This sentence needs to be revised: “The aim of the study was to characterize individual patient gene expression profiles and to identify the relationship with clinical outcomes”. They have done mutational analysis.*

**Reply:** As suggested by the reviewer, we have revised this sentence (line 34).

*Need some references in Introduction section: Importantly, multiple studies suggest that the prevalence of TNBC among HL women can be slightly higher compared to NHW, approaching 23.1%. Also, the onset of the disease occurs at age approximately 11 years younger than the average age reported for NHW and AA women.*
**Reply:** As suggested by the reviewer, we have added additional references [12-14], and [1, 11] to support our statement.

*Methods: It is mentioned that patients were stage II-IV. Please confirm metastatic status of all samples. Please clarify the rationale of selecting 24 HL out of 25 TNBC patients. Describe the control samples used for this study.*

**Reply:** In this study, we included patients with newly diagnosed stage IV (metastatic) TNBC, as well patients who were initially diagnosed with stages II–III and who progressed to stage IV during the 12-month observation period after the definitive treatment (surgery, chemotherapy, and radiation therapy). We did not have any control samples in the study because the original study was designed as a retrospective study without any interventions. We only compared HL patients with TNBC from national and international databases. We analyzed the tumor mutation profile of all 25 patients and highlighted that one patient was not HL.

*Results:
Authors described Notch1-3. How about Notch 4? In the discussion section, authors talked about the role of Notch 4 from another paper.*

**Reply:** We did not detect any *NOTCH4* mutations in our patient population.

*Table: 1
Tell us about the effect of Immunotherapy treatments. Which immunotherapy was used and which combinations?*

**Reply:** Since we did not observe any statistically significant difference in PFS or OS following immunotherapy, and also because the sample size was too small for further analyses, we decided to remove this information from Table 1. For eligible patients, we used a standard of care treatment protocol (IMpassion 130 randomized phase III clinical trial) approved by the FDA on 03/08/2019: combined chemotherapy nab-paclitaxel 100 mg/m^2^ D1;8;15 Q 21 days cycle with anti-PDL-1 agent atezolizumab 840 mg D1;15 Q21 days(line 174).

*Table 3: Rationale for the three clusters.*

**Reply:** We performed a variable cluster analysis for categorical variables and determined the number of clusters using an aggregation plot and mean adjusted Rand criteria, which indicated that three clusters retained maximum variability in the data based on the genes. This was a very useful analysis in this study, as several mutated genes might interact with each other. By cluster analysis, we were able to evaluate the joint effect of two or more gene mutations on overall survival and progression-free survival. We have added this information to the revised manuscript(lines 157–160).

*Discussion: We all know the importance of p53 in TNBC and other breast cancers. This manuscript talks extensively on Notch mutations. I would like to see the Notch expression compared to other HL patients in the cited paper (17 and 25).*
**Reply**: Thank you for this important point. However, in our study, we did not perform gene expression analysis. Therefore, we were unable to compare our data with published studies. Furthermore, we did not find any data for Notch expression in Hispanic or Asian patients in Jiang Z et al. (Genomic and Transcriptomic Landscape of Triple-Negative Breast Cancers: Subtypes and Treatment Strategies.) (ref 17). In addition, in Wang K et al., while the authors described activating mutations in *NOTCH1*, *NOTCH2*, and *NOTCH3* and extensively discussed *in vitro* experimental data as well performing as some comparisons with TCGA database, they did not analyze and/or discuss any data for HL.

*Many GSI related clinical trials have been postponed or terminated. Please address those as well. One of the major issues of GSI is that it causes severe intestinal toxicity; more importantly, Notch is required for T cell functions as well.*
**Reply**: We agree with your remark, and have added a relevant sentence dealing with this issue to the revised manuscript (line 409). We also decided to remove the discussion about Notch inhibition in clinical trials. Unfortunately, the results of multiple phase I trials showed some disappointments of Notch pathway inhibition. These issues were not only because of GI toxicity but also because of low response rates.

Furthermore, among multiple previous studies, only a few showed some efficacy of Notch inhibition. In 2019, the FDA granted orphan drug designation to AL101 for the treatment of patients with adenoid cystic carcinoma with activating mutations in Notch. A phase II study (ACCURACY) is still recruiting patients.

*Spelling check:*

*Innovatibe,datat*
**Reply:** The suggested corrections have been made and a draft of the revised manuscript has been edited by a native English editor.

*Need references: “Nevertheless, we identified two smaller studies (n=19) from Northeast Mexico, and another study from the National Cancer Institute of Mexico in Mexico City (n=12) analyzing data from a similar population of patients”.*

**Reply:** As suggested by the reviewer, we have added these references to the revised manuscript ([29] and [30]).
